# A Fatal Case of Acute Pulmonary Embolism after Cyanoacrylate Closure for Varicose Veins

**DOI:** 10.3400/avd.cr.25-00113

**Published:** 2026-03-01

**Authors:** Kenichi Chatani, Hiroyuki Ihori, Kazumasa Ohara, Makoto Nonomura, Tomoki Kameyama, Hiroshi Inoue

**Affiliations:** Department of Cardiology, Saiseikai Toyama Hospital, Toyama, Toyama, Japan

**Keywords:** varicose vein, cyanoacrylate closure, pulmonary embolism

## Abstract

An 81-year-old female with bilateral small saphenous vein varicosities (CEAP, C2s, Ep, As, Pr) underwent cyanoacrylate closure (CAC) and stab avulsion under general anesthesia. Fourteen days later, she developed severe dyspnea and was diagnosed with pulmonary embolism (PE) and deep vein thrombosis. Despite anticoagulation, cardiopulmonary support, and catheter-directed thrombectomy, she died 24 days after admission. Adequate heparin dosing with activated partial thromboplastin time monitoring is important. Prolonged procedures under general anesthesia may increase PE risk. Early ambulation, compression therapy, and follow-up ultrasonography beyond 24 hours may help detect delayed thrombus formation and reduce life-threatening complications after CAC.

## Introduction

Cyanoacrylate closure (CAC) is a highly effective strategy for treating varicose veins and chronic venous insufficiency. CAC is an endovenous embolization therapy that utilizes N-butylcyanoacrylate glue and is characterized as a non-thermal, non-tumescent, minimally invasive procedure with a favorable safety profile. In Japan, CAC was approved for clinical use in December 2019.

Although only a few reports have described cases of deep vein thrombosis (DVT) or pulmonary embolism (PE) following CAC, no fatal cases have been reported to date. Here, we report a rare case of fatal PE that developed 2–14 days after CAC for varicose veins of the bilateral small saphenous veins (SSVs) under general anesthesia.

## Case Report

**Fig. 1:**
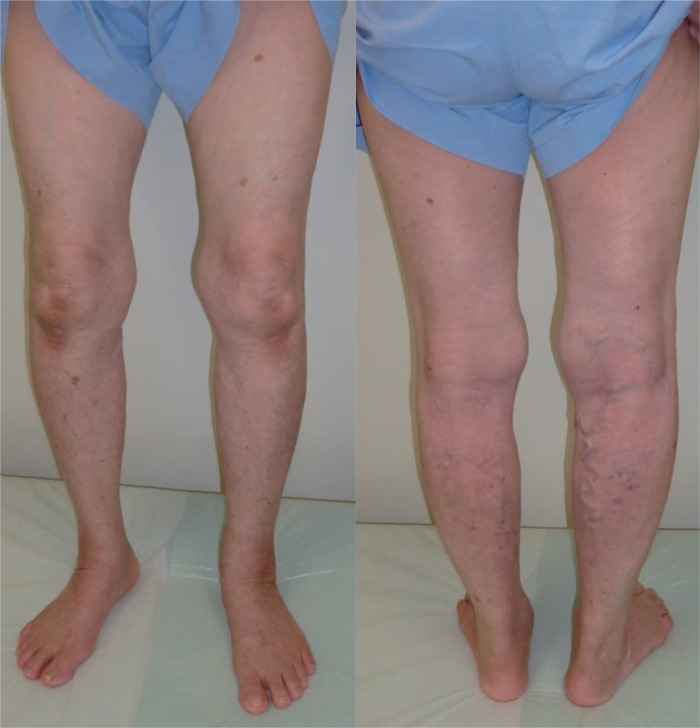
Superficial varicose veins (CEAP, C2s, Ep, As, Pr, SSV) observed on both legs. SSV: small saphenous vein

An 81-year-old female with a medical history of hyperlipidemia, hypertension, and type 2 diabetes mellitus presented with bilateral superficial varicose veins associated with dullness (CEAP classification: C2s, Ep, As, Pr, and SSV for both sides; **Fig. 1**). Lower extremity venous ultrasonography did not reveal thrombus in the superficial or deep veins of the lower legs, including the saphenopopliteal junction (SPJ), but did reveal valve insufficiency in the bilateral SSVs. Her daily medications included bezafibrate 400 mg, lansoprazole 15 mg, and ursodeoxycholic acid 600 mg. She had a body mass index of 20.8 kg/m^2^ and no history of allergies or malignancy. Compression stocking therapy was initially performed, but she discontinued use on 1 leg due to difficulty in application.

One month later, she underwent CAC using VenaSeal Closure System (Medtronic, Minneapolis, MN, USA) for bilateral SSVs, as well as stab avulsion for varicose veins in the right lower calf; the procedure lasted 75 min at another clinic. CAC for bilateral SSVs required 40 min, and stab avulsion required 15 min. The treatment was performed under general anesthesia without endotracheal intubation at the patient’s request. Cyanoacrylate glue was injected 5 times along an 18-cm segment of the right SSV and 4 times along a 15-cm segment of the left SSV via proximal calf punctures, followed by stab avulsion for varicose veins of the right lower calf. All procedures were performed in the prone position, and elastic bandages were applied postoperatively. She began ambulation about 10 minutes after the procedure and was subsequently discharged the same day.

On the day following the procedure, lower extremity venous ultrasonography revealed no evidence of endovenous glue-induced thrombosis (EGIT) in the SPJ, DVT, or recanalization of the treated veins at the outpatient visit. Seven days after CAC, she began experiencing palpitations and exertional dyspnea and visited the clinic, but specific tests were not performed. On day 14 post-procedure, she presented with severe dyspnea and was admitted to our hospital by referral with a suspected diagnosis of PE.

**Fig. 2:**
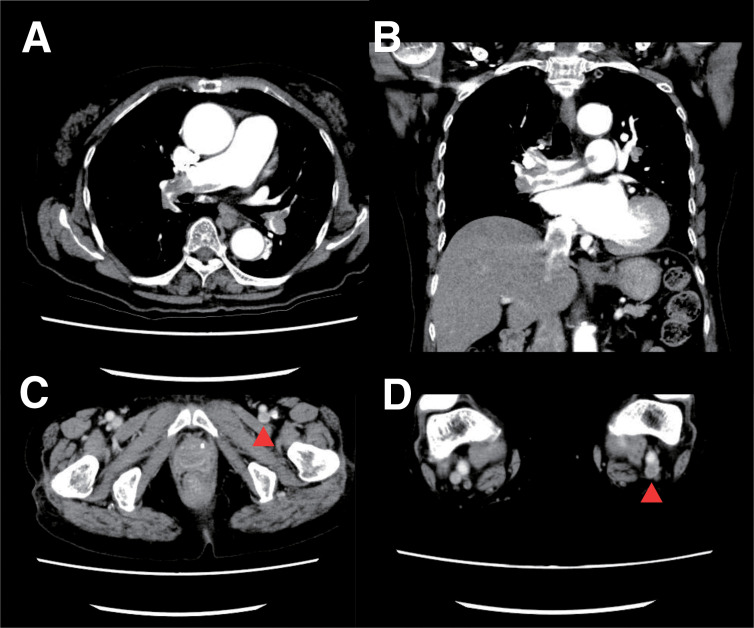
Contrast-enhanced computed tomography performed 14 days after CAC. (**A**) Massive thrombi are present in the bilateral pulmonary arteries (short-axis view). (**B**) Massive thrombi are present in the bilateral pulmonary arteries (long-axis view). (**C**) Deep vein thrombosis was detected in the left common femoral vein (indicated by the red arrowhead). (**D**) Thrombi were detected in the left popliteal vein (indicated by the red arrowhead). Superficial vein thrombi were also identified in bilateral small saphenous veins (not shown in this slice). CAC: cyanoacrylate closure

On admission, her pulse rate was 84 bpm (regular); blood pressure, 126/84 mmHg; and oxygen saturation, 92% on 5 L/min oxygen via face mask. Laboratory data showed elevated levels of D-dimer (7.2 μg/mL) and B-type natriuretic peptide (217.7 pg/mL). Coagulation studies, including antithrombin III, protein C, protein S, plasminogen, fibrinogen, antinuclear antibody, and anticardiolipin antibody, revealed no abnormalities predisposing to thrombosis. Electrocardiogram showed sinus rhythm with T-wave inversion in leads I, aVL, II, III, aVF, and V1–V6. Transthoracic echocardiography revealed right ventricular dilatation and moderate tricuspid regurgitation with a pressure gradient of 43.3 mmHg, indicating pulmonary hypertension. Contrast-enhanced computed tomography demonstrated massive pulmonary emboli in the pulmonary trunk and bilateral pulmonary arteries, as well as thrombi in the left common femoral vein, left popliteal vein, and both SSVs (**Fig. 2**). Leg vein ultrasonography demonstrated superficial vein thrombi in both SSVs and deep vein thrombi in the left common femoral vein and popliteal vein. Upon admission, a 5000-unit intravenous bolus of unfractionated heparin was administered, followed by continuous infusion at a rate of 12000 units per day. Unfortunately, activated partial thromboplastin time (APTT) was not measured for monitoring. On the morning of the second day of hospitalization, she developed sudden loss of consciousness and cardiovascular collapse. Percutaneous cardiopulmonary support (PCPS) and intra-aortic balloon pumping were initiated, and catheter-directed thrombectomy was performed.

Her hemodynamic status was stabilized without administration of catecholamines 5 days after PCPS introduction. On day 7 post-PCPS introduction, transthoracic echocardiography revealed mild tricuspid regurgitation with a pressure gradient of 33.8 mmHg, indicating improvement of right ventricular function and pulmonary hypertension. On day 8 post-PCPS introduction, PCPS flow was reduced to less than 1.0 L/min. She was successfully weaned from mechanical support 12 days after PCPS introduction. However, her blood pressure tended to drop due to septic shock from day 17 post-PCPS introduction. She died 22 days after PCPS introduction due to acute respiratory distress syndrome, pneumonia, and sepsis without regaining consciousness. Autopsy was not performed because consent could not be obtained from her family.

## Discussion

Anticoagulant therapy for acute PE consists of continuous infusion of unfractionated heparin, with the goal of achieving an adequate heparin effect (APTT level 1.5–2.5 times the control value) during the initial 24 hours following a 5000-unit intravenous bolus.^1)^ The indication of inferior vena cava (IVC) filter is controversial, but severe PE with right heart overload and central DVT is a good indication. In this patient, a 5000-unit intravenous bolus of unfractionated heparin was administered, followed by continuous infusion at a rate of 12000 units per day. APTT was not measured for monitoring. An IVC filter was not implanted because the right heart load was mild on echocardiography and DVT did not exist in the iliac vein on admission. The reason for the sudden change in this patient may have been the small dose of continuous unfractionated heparin.

There have been several reports of DVT and PE occurring after CAC.^2)^ In most cases, DVT occurs within 24 hours postoperatively. In our case, venous ultrasonography performed 1 day after CAC revealed no evidence of EGIT, DVT, or recanalization. However, the patient developed PE between 2 and 14 days after the procedure. This suggests that thrombus formation could occur after the initial 24-hour window and subsequently lead to PE. Follow-up leg vein ultrasonography on the day of and 2 days following the procedure could help detect hypercoagulable conditions after CAC.

Although post-procedural compression stockings are generally not required after CAC, they have been shown to reduce the risk of DVT. Our patient did not wear compression stockings after the procedure. Given that the surgery was performed under general anesthesia, a known risk factor for venous thromboembolism, postoperative compression therapy may have been beneficial in preventing DVT or PE in our patient.

Surgical procedures under general anesthesia inherently carry a higher risk of DVT and PE, though the degree of risk varies with the type of procedure. Several studies have shown a low incidence of DVT and PE following venous surgeries.^3)^ For example, Miller et al. reported incidence rates of 0.4% for DVT and 0.1% for PE after varicose vein surgery.^4)^ Similarly, the incidence of DVT and PE after endovenous therapies, including radiofrequency ablation, endovenous laser ablation (EVLA), and CAC, remains very low.^1,5,6)^ Reported incidences of DVT and PE after CAC are 0.18% and 0.01%, respectively^5)^; the incidence of PE after EVLA was only 0.0067%.^6)^ However, there is at least 1 case report of death due to PE following EVLA under general anesthesia.^7)^

The mechanism of PE in the present case remains uncertain. However, it is possible that DVT was caused by a combination of endothelial dysfunction due to catheter manipulation, venous stasis, and a hypercoagulable state following CAC. Additionally, superficial vein thrombosis may have propagated into the deep venous system, ultimately leading to PE. Several studies have identified risk factors for DVT and PE following endovenous thermal ablation (ETA), including advanced age, prior history of DVT or superficial thrombophlebitis, and elevated D-dimer levels without concurrent C-reactive protein elevation.^8,9)^

DVT has also been shown to occur more frequently when stab avulsion is performed simultaneously with endovenous therapy, compared to ETA alone.^10)^

Our patient underwent CAC for bilateral SSVs along with stab avulsion of the right lower leg in the prone position under general anesthesia lasting 75 min. Endovenous therapy is typically performed under local or intravenous sedation. Prolonged general anesthesia is relatively uncommon and might have increased thrombotic risk in our patient. After CAC, especially under general anesthesia, early postoperative ambulation and continued ambulation could be important to prevent venous thromboembolism.^6)^

Taken together, the likely contributors to DVT and subsequent PE in our patient include the relatively long operative time and simultaneous procedures under general anesthesia. When CAC is performed under general anesthesia, it may be advisable to implement prophylactic measures such as compression stockings or intermittent pneumatic compression devices postoperatively, as well as early postoperative ambulation. In addition, follow-up leg vein ultrasonography more than 24 hours after the procedure could help detect delayed thrombus formation and potentially prevent life-threatening complications.

## Conclusion

We report a rare fatal case of PE that developed 2–14 days after CAC and stab avulsion for varicose veins of the bilateral SSVs under general anesthesia. When CAC is performed under general anesthesia, early postoperative ambulation, compression therapy, and follow-up leg vein ultrasonography beyond 24 hours after the procedure should be considered for prevention and detection of thromboembolic complications.
